# Effectiveness of rectal artesunate as pre-referral treatment for severe malaria in children under 5 years of age: a multi-country observational study

**DOI:** 10.1186/s12916-022-02541-8

**Published:** 2022-10-11

**Authors:** Manuel W. Hetzel, Jean Okitawutshu, Antoinette Tshefu, Elizabeth Omoluabi, Phyllis Awor, Aita Signorell, Nina C. Brunner, Jean-Claude Kalenga, Babatunde K. Akano, Kazeem Ayodeji, Charles Okon, Ocheche Yusuf, Proscovia Athieno, Joseph Kimera, Gloria Tumukunde, Irene Angiro, Giulia Delvento, Tristan T. Lee, Mark J. Lambiris, Marek Kwiatkowski, Nadja Cereghetti, Theodoor Visser, Harriet G. Napier, Justin M. Cohen, Valentina Buj, Christian Burri, Christian Lengeler

**Affiliations:** 1 Swiss Tropical and Public Health Institute, Allschwil, Switzerland; 2 University of Basel, Basel, Switzerland; 3 Kinshasa School of Public Health, Kinshasa, Democratic Republic of the Congo; 4 Akena Associates, Abuja, Nigeria; 5 Makerere University School of Public Health, Kampala, Uganda; 6 Clinton Health Access Initiative, Boston, MA USA; 7 UNICEF, New York, NY USA

**Keywords:** Severe malaria, Malaria treatment, Rectal artesunate, Referral, Child mortality, Malaria mortality, Case management, Community health worker

## Abstract

**Background:**

To prevent child deaths from severe malaria, early parenteral treatment is essential. Yet, in remote rural areas, accessing facilities offering parenteral antimalarials may be difficult. A randomised controlled trial found pre-referral treatment with rectal artesunate (RAS) to reduce deaths and disability in children who arrived at a referral facility with delay. This study examined the effectiveness of pre-referral RAS treatment implemented through routine procedures of established community-based health care systems.

**Methods:**

An observational study accompanied the roll-out of RAS in the Democratic Republic of the Congo (DRC), Nigeria and Uganda. Children <5 years of age presenting to a community-based health provider with a positive malaria test and signs of severe malaria were enrolled and followed up during admission and after 28 days to assess their health status and treatment history. The primary outcome was death; covariates of interest included RAS use, referral completion, and post-referral treatment.

**Results:**

Post-roll-out, RAS was administered to 88% of patients in DRC, 52% in Nigeria, and 70% in Uganda. The overall case fatality rate (CFR) was 6.7% (135/2011) in DRC, 11.7% (69/589) in Nigeria, and 0.5% (19/3686) in Uganda; 13.8% (865/6286) of patients were sick on day 28. The CFR was higher after RAS roll-out in Nigeria (16.1 vs. 4.2%) and stable in DRC (6.7 vs. 6.6%) and Uganda (0.7 vs. 0.3%). In DRC and Nigeria, children receiving RAS were more likely to die than those not receiving RAS (aOR=3.06, 95% CI 1.35–6.92 and aOR=2.16, 95% CI 1.11–4.21, respectively). Only in Uganda, RAS users were less likely to be dead or sick at follow-up (aOR=0.60, 95% CI 0.45–0.79). Post-referral parenteral antimalarials plus oral artemisinin-based combination therapy (ACT), a proxy for appropriate post-referral treatment, was protective. However, in referral health facilities, ACT was not consistently administered after parenteral treatment (DRC 68.4%, Nigeria 0%, Uganda 70.9%).

**Conclusions:**

Implemented at scale to the recommended target group, pre-referral RAS had no beneficial effect on child survival in three highly malaria-endemic settings. RAS is unlikely to reduce malaria deaths unless health system issues such as referral and quality of care at all levels are addressed.

**Trial registration:**

The study is registered on ClinicalTrials.gov: NCT03568344.

**Supplementary Information:**

The online version contains supplementary material available at 10.1186/s12916-022-02541-8.

## Background

Of the estimated 627,000 annual malaria deaths, the majority (ca. 80%) occur in children under 5 years of age living in sub-Saharan Africa [1]. Progress towards further reducing the death toll from malaria has stalled in recent years, and new and complementary approaches are needed to accelerate towards global and national malaria elimination targets [1]. Access to prompt treatment with parenteral artesunate followed by a full course of an oral artemisinin-based combination therapy (ACT) alongside the management of complications can save a child suffering from a life-threatening episode of severe malaria [2].

Usually, only secondary or tertiary level health facilities have inpatient wards with the capacity to manage a child with severe malaria comprehensively. Primary health centres (PHC) are often neither equipped nor allowed to administer parenteral antimalarials. Community health workers (CHW) are trained to administer oral treatment for uncomplicated malaria episodes but are not qualified to diagnose or treat severe malaria. Integrated Community Case Management (iCCM) algorithms direct CHWs to identify children with danger signs of severe illness (including those indicative of severe malaria) and then immediately refer the child to the nearest health facility [3]. Children are more likely to suffer from fatal or debilitating consequences of severe malaria in areas where higher-level health facilities are difficult to access [4, 5].

At the primary health care level, options to manage severe malaria are typically limited to pre-referral treatment. The World Health Organization (WHO) recommends a single dose of intramuscular artesunate, or, if injections are not available and the patient is under 6 years of age, a single rectal dose of artesunate, followed by immediate referral to a higher-level health facility [2]. Rectal artesunate (RAS) rapidly reduces the malaria parasite load [6] and in a randomised placebo-controlled trial conducted in Bangladesh, Ghana and Tanzania, pre-referral RAS was found to reduce case fatality of an episode of broadly defined severe malaria by 26% (risk ratio [RR] 0.74, 95% CI 0.59–0.93) in children below 6 years of age [7, 8]. In children who took more than 6 h to reach a higher-level facility, RAS reduced deaths and permanent disability by about 50% (RR 0.49, 95% CI 0.32–0.77) [7].

Evidence from a randomised controlled trial is unlikely to reflect the real-world effect of rolling out RAS at scale and offers limited operational guidance on the optimal way of introducing RAS [9]. The relationship between treatment coverage and health impact at a population level is complex and is affected by the broader health system context [10]. Previously, the unavailability of a WHO prequalified RAS product hampered the generation of real-world evidence of the effectiveness of RAS. With two RAS formulations having obtained prequalification since 2018 and an increasing number of malaria control programmes ordering RAS [11, 12], there was an urgent need to generate evidence of the impact of introducing pre-referral RAS in routine clinical practice. The potential of RAS to prevent malaria deaths in high-burden settings must be ascertained to establish the role of this intervention in the quest to reduce malaria mortality by 90% by 2030 [13].

Here, we report on the principal findings of the Community Access to Rectal Artesunate for Malaria (CARAMAL) Project, an observational study accompanying the large-scale introduction of pre-referral quality-assured RAS in established community-based health care systems in the Democratic Republic of the Congo (DRC), Nigeria, and Uganda.

## Methods

### Study design

This was an observational study accompanying the roll-out of pre-referral RAS delivered by CHWs implementing iCCM and health workers at PHCs. The research team conducted the investigations independently; the training of health care providers, behaviour change and communication activities, and continuous supply of RAS was the responsibility of local health authorities with support from UNICEF. Data collection started 8–10 months before RAS roll-out and covered 15–17 months of the post-roll-out period.

### Setting

The study was conducted in three Health Zones (Ipamu, Kenge and Kingandu) in DRC, three Local Government Areas (Fufore, Mayo-Belwa and Song) of Adamawa State in Nigeria, and three districts (Kole, Kwania and Oyam) in Uganda. The total population of the study areas was approximately 2.5 million people, including 476,000 (19%) children under 5 years of age (Additional file 1: Table S1). Curative health services in the study areas were provided by CHWs implementing iCCM, PHCs, and referral health facilities. Further details of the study sites can be found elsewhere [14].

### Participants and procedures

Children under 5 years of age presenting to a CHW or PHC with a history of fever plus at least one general danger sign according to national iCCM guidelines (Additional file 1: Table S1), were provisionally enrolled in the study. These enrolment criteria conform with iCCM criteria for administering RAS. A malaria rapid diagnostic test (mRDT) was performed for study purposes as current iCCM algorithms do not include malaria testing of children with danger signs. Health care providers reported each provisional enrolment to the local study coordinator, who included the patient in the study database and scheduled a follow-up visit 28 days after provisional enrolment. Reporting procedures included regular pro-active contacts between the study team and enrolling CHWs and staff at PHCs, during which patient characteristics and information on RAS administration were extracted from provider records. Day 28 visits were carried out at the patient’s home by trained study staff. The home visits included structured interviews with the patient’s caregiver to record the patient’s health status on the day of the visit and retrospectively elicit the history of signs and symptoms, treatment-seeking behaviour, and administration of antimalarials including RAS. Study participants who were sick during the home visits were referred to the nearest health facility. For deceased children, the caregiver interviews which included details on the circumstances of death were postponed for up to one month to respect the mourning period. During follow-up, an mRDT (CareStart or SD Bioline, brand as per local routine clinical practice) was performed and haemoglobin concentration was measured (HemoCue hand-held photometer) on capillary blood collected from a single finger or heel prick. Patients who were successfully referred from a CHW or PHC to the main referral health facilities in the study areas were monitored during their admission. Procedures and outcomes during admission were recorded by study nurses based at the referral facility.

Data was collected on tablets using structured electronic forms in ODK Collect (https://opendatakit.org/). The secure ODK Aggregate server was hosted at the Swiss Tropical and Public Health Institute.

CHWs and PHC staff were sensitised and trained about the study during dedicated workshops. All study staff members underwent extensive training on study purpose, informed consent administration, and field data collection procedures.

### Outcomes

Death from any cause within 28 days of provisional enrolment was the primary study outcome. A secondary outcome was caregiver-reported illness of the child on day 28 (including both acute episodes and ongoing illness); mRDT-positivity and haemoglobin (Hb) concentration on day 28 were complementary indicators of morbidity. Exposures of interest included RAS use completed referral to a referral health facility with inpatient ward (Additional file 1: Table S1), treatment with a parenteral antimalarial, and treatment with an artemisinin-based combination therapy. In DRC and Nigeria, the analysis only accounted for antimalarial treatment administered at referral health facilities where CARAMAL staff were present. In Uganda, information provided by the caregiver on antimalarial treatment obtained from any healthcare provider was taken into consideration as study staff could not monitor all facilities that provided post-referral treatment.

### Statistics

The severe malaria case fatality rate (CFR) pre-RAS was assumed to be 6% based on several controlled trials [15, 16]; thus a minimum of 6032 patients was required to detect a 30% reduction in CFR between a 6-month pre-RAS and 18-month post-RAS period with 80% power and α = 0.05 [17].

The analyses included enrolled patients with an acute fever or history of fever, at least one iCCM general danger sign (as per national guidance) as recorded by the enrolling provider and/or reported during the home visit interview (Additional file 1: Table S1), a positive mRDT at provisional enrolment, and successful follow-up 28 days after provisional enrolment. Deaths were considered up to 3 days after the official day 28.

Outcomes were calculated for each country as overall proportions, for the pre-RAS and post-RAS periods, and for RAS-users and non-users. Proportions were compared by chi-square test. Country-specific logistic regression models estimated the unadjusted and adjusted association of RAS use with the day 28-outcomes ‘dead’ and the composite outcome ‘dead or sick’. The enrolling provider or a village proxy were included in all models as a random effect. The set of adjustments varied between countries depending on the local context and the number of events available to analyse. Details are provided in the footer of Table 4. Information on RAS administration is based on consolidated health worker records and caregiver reports.

Data analysis was performed in Stata/SE 15.1 and 16.1 (StataCorp, College Station, TX, USA).

## Results

### Participants

Between April 2018 and July 2020, 8365 patients were provisionally enrolled by a CHW or at a PHC. Of these, 772 (9%) were not followed-up or did not provide informed consent, 365 (4%) had no record of a positive mRDT, and 942 (11%) did not fulfil all inclusion criteria. Hence, 6286 patients were included in this analysis, of whom 3402 were treated with RAS (Additional file 2: Figure S1). Patients in Nigeria were on average older and more frequently male than in the other countries. Age and sex distributions did not differ between RAS users and non-users, except in DRC, where RAS users were slightly older (Table 1). The frequency of individual danger signs reported at enrolment differed between the three countries and, in some instances, between RAS users and non-users. Convulsions, often a sign of cerebral involvement [18], were more common among those children who received RAS. In DRC, a majority of patients were enrolled at PHCs; in Uganda, enrolments were exclusively from CHWs.

**Table 1:** Study patient characteristics by country and rectal artesunate use

Background characteristic	DRC			Nigeria			Uganda		
	No RAS (***N***=475)	RAS (***N***=1536)	***P***-value	No RAS (***N***=391)	RAS (***N***=198)	***P***-value	No RAS (***N***=2018)	RAS (***N***=1668)	***P***-value
Female, *n* (%)	221 (47)	719 (47)	0.91	151 (39)	86 (43)	0.26	944 (47)	781 (47)	0.98
Mean age in years (SD)	1.6 (1.3)	1.8 (1.3)	0.002	2.0 (1.2)	1.9 (1.2)	0.67	1.8 (1.3)	1.8 (1.2)	0.55
Danger sign at enrolment, *n* (%)									
Convulsions	248 (52)	884 (58)	0.04	233 (60)	154 (78)	< 0.001	590 (29)	829 (50)	< 0.001
Unusually sleepy/unconscious	175 (37)	322 (21)	< 0.001	249 (64)	119 (60)	0.40	1239 (61)	1480 (89)	< 0.001
Not able to drink or feed	322 (68)	704 (46)	< 0.001	252 (64)	107 (54)	0.01	1152 (57)	1262 (76)	< 0.001
Vomiting everything	44 (9)	160 (10)	0.47	284 (73)	103 (52)	< 0.001	1271 (63)	1038 (62)	0.64
Enrolment location, *n* (%)									
Community health worker	21 (4)	69 (4)		227 (58)	87 (44)		2018 (100)	1668 (100)	
Primary health centre	454 (96)	1467 (96)	0.95	164 (42)	111 (56)	0.001			
Area (DRC/Nigeria/Uganda), *n* (%)			< 0.001			< 0.001			< 0.001
Ipamu/Fufore/Kole	81 (17)	556 (36)		185 (47)	52 (26)		1303 (65)	410 (25)	
Kenge/Mayo-Belwa/Oyam	207 (44)	536 (35)		150 (38)	99 (50)		398 (20)	576 (35)	
Kingandu/Song/Kwania	187 (39)	444 (29)		56 (14)	47 (24)		317 (16)	682 (41)	
Rainy season^a^, *n* (%)	342 (72)	738 (48)	< 0.001	281 (72)	159 (80)	0.03	1441 (71)	868 (52)	< 0.001
RAS implementation period, *n* (%)									
Pre-RAS	302 (64)	2 (0)		217 (55)	0 (0)		1394 (69)	47 (3)	
Post-RAS	173 (36)	1534 (100)	< 0.001	174 (45)	198 (100)	< 0.001	624 (31)	1621 (97)	< 0.001

*RAS* Rectal artesunate

^a^DRC: October–April; Nigeria: May–October; Uganda: April–October

### Rectal artesunate intervention and continuum of care

**Fig. 1:**
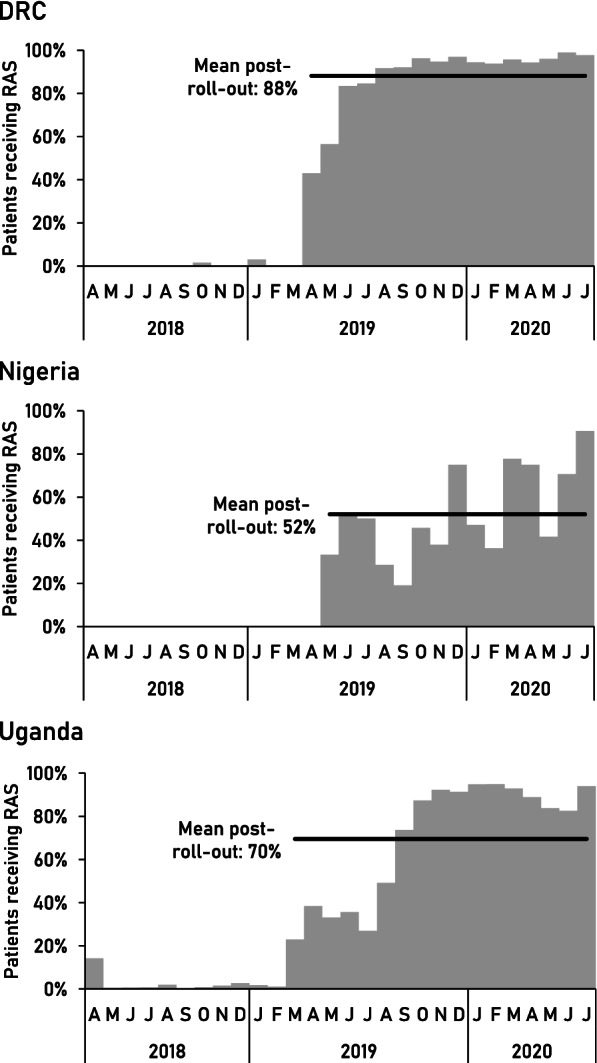
RAS use among study patients, by month

RAS distribution to CHWs and PHCs started between March and April 2019 through existing local supply chain mechanisms, as described in detail elsewhere [14]. Thereafter, RAS was administered, on average, to 88% of study patients in DRC, 52% in Nigeria, and 70% in Uganda. Coverage fluctuated strongly in Nigeria and uptake was slow in the first six months in Uganda (Fig. 1).

Treatment and referral patterns differed between the countries (Table 2). Study children in Nigeria were least likely to complete referral. Most children who completed referral did so within one day. In the referral facilities monitored by study nurses, post-referral treatment with parenteral antimalarials followed by an oral ACT was not universally practised. None of the study children in Nigeria received an ACT prior to their discharge from the referral facility. Further details on referral completion and post-referral treatment in the study area are available elsewhere [19, 20].

**Table 2:** Treatment and referral along the continuum of care for the entire study period

Exposure variables	DRC	Nigeria	Uganda
**RAS use (*N*)**	**2011**	**589**	**3686**
Yes (%)	76.4	33.6	45.3
**Referral completion to referral health facility (*N*)**	**2011**	**589**	**3686**
Yes (%)	65.0	41.3	57.3
Unconfirmed (%)	2.4	12.9	0.6
**Referral delay (*N*)**	**2011**	**589**	**3686**
Same or next day (%)	47.7	33.1	50.1
Later than the next day (%)	14.9	5.6	4.6
Not completed (%)	32.5	45.8	42.1
Unknown (%)	4.9	15.5	3.2
***Post-referral treatment***^a^			
**Artesunate injection (*N*)**	**1215**	**177**	**1420**
Yes (%)	78.7	94.4	43.7
**Antimalarial injection**^b^ **(*N*)**	**1215**	**177**	**1436**
Yes (%)	86.8	94.4	51.5
**ACT (*N*)**	**1215**	**177**	**2111**
Yes (%)	68.4	0	70.9
**Antimalarial injection**^b^ **and ACT (*N*)**	**1215**	**177**	**1351**
Yes (%)	63.8	0	46.0

*RAS* Rectal artesunate, *ACT* Artemisinin-based combination therapy

^a^For DRC and Nigeria, observed practice for patients who completed referral and were admitted at monitored referral health facilities; for Uganda, as per caregiver recall on day 28, which may include medicines administered outside monitored referral facilities (denominator includes caregivers who recall whether or not a specific medicine was administered)

^b^Includes parenteral artesunate, artemether and quinine

### Health status at day 28 follow-up

#### Death

**Fig. 2:**
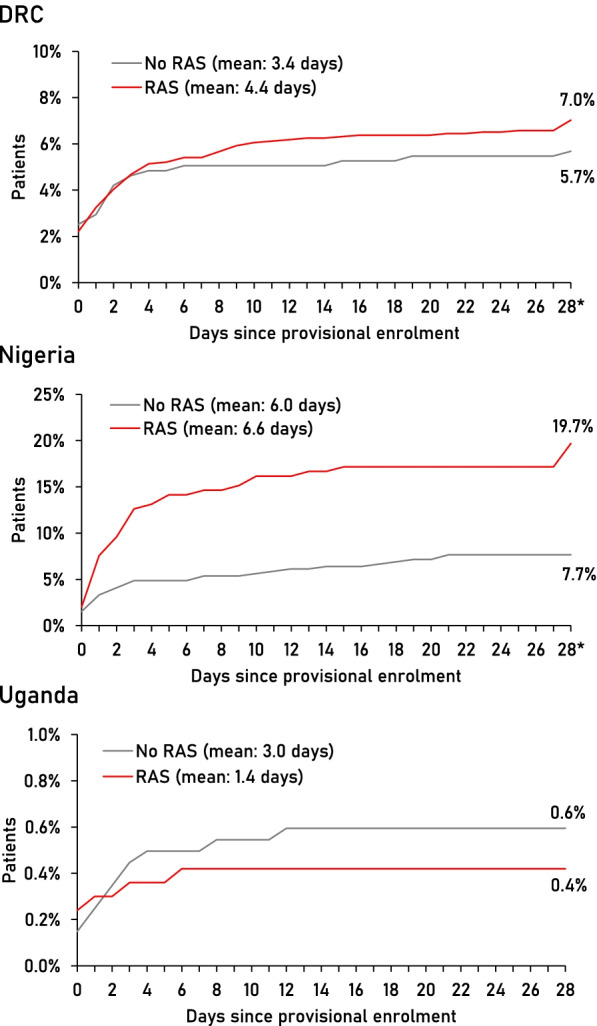
Time to death in days since provisional enrolment (provider attendance) of study participants, by country and RAS use. Note difference in *y*-axis scales. *Deaths up to day 31 were considered and included in this time point

A total of 223 deaths were registered among the study patients (135 in DRC, 69 in Nigeria, 19 in Uganda). The overall CFR was 6.7% (135/2011) in DRC, 11.7% (69/589) in Nigeria, and 0.5% (19/3686) in Uganda (*p* < 0.001) (Table 3). None of the deaths was from an accident or injury. Most deaths occurred in a health facility (DRC 73%, Nigeria 49%, Uganda 42%), on the way to a (referral) health facility (DRC 17%, Nigeria 10%, Uganda 26%), and at home (DRC 7%, Nigeria 35%, Uganda 32%). In DRC and Nigeria, deaths occurred over the entire follow-up period (Fig. 2). Deaths occurred later in Nigeria (mean 6.3 days after provisional enrolment) than in DRC (4.2 days) and Uganda (2.4 days) (*p* = 0.03). While there was no significant change in CFR after the roll-out of RAS in DRC and Uganda (Table 3), an increase was observed in Nigeria among both CHW and PHC enrolments (RR = 3.5, 95% CI 1.2–10.3 and 2.6, 95% CI 1.1–6.3, respectively).

**Table 3:** Health outcomes at day 28 follow-up by country, RAS implementation phase, and RAS use

	DRC			Nigeria			Uganda			Between-country ***p***-value
	***n***/***N***	(%)	***P***-value*	***n***/***N***	(%)	***P***-value*	***n***/***N***	(%)	***P***-value*	
**Case fatality rate**										
Overall	135/2011	(6.7)		69/589	(11.7)		19/3686	(0.5)		<0.001
*Implementation*										
Pre-RAS	20/304	(6.6)		9/217	(4.2)		4/1441	(0.3)		
Post-RAS	115/1707	(6.7)	0.92	60/372	(16.1)	<0.001	15/2245	(0.7)	0.14	
*RAS use*										
No	27/475	(5.7)		30/391	(7.7)		12/2018	(0.6)		
Yes	108/1536	(7.0)	0.34	39/198	(19.7)	<0.001	7/1668	(0.4)	0.45	
**Sick at day 28 follow-up**										
Overall	242/2011	(12.0)		34/589	(5.8)		589/3686	(16.0)		0.002
*Implementation*										
Pre-RAS	40/304	(13.2)		20/217	(9.2)		299/1441	(20.8)		
Post-RAS	202/1707	(11.8)	0.59	14/372	(3.8)	0.007	290/2245	(12.9)	0.003	
*RAS use*										
No	72/475	(15.2)		25/391	(6.4)		428/2018	(21.2)		
Yes	170/1536	(11.1)	0.04	9/198	(4.6)	0.30	161/1668	(9.7)	<0.001	
**mRDT-positive at day 28 follow-up**										
Overall	811/1843	(44.0)		256/510	(50.2)		2547/3667	(69.5)		<0.001
*Implementation*										
Pre-RAS	159/284	(56.0)		97/208	(46.6)		953/1437	(66.3)		
Post-RAS	652/1559	(41.8)	<0.001	159/302	(52.7)	0.27	1594/2230	(71.5)	0.02	
*RAS use*										
No	235/444	(52.9)		168/357	(47.1)		1405/2006	(70.0)		
Yes	576/1399	(41.2)	<0.001	88/153	(57.5)	0.03	1142/1661	(68.8)	0.44	
**Severe anaemia (Hb < 7 g/dL) at day 28 follow-up**										
Overall	65/1875	(3.5)		33/514	(6.4)		151/3379	(4.5)		0.054
*Implementation*										
Pre-RAS	14/284	(4.9)		21/208	(10.1)		69/1256	(5.5)		
Post-RAS	51/1591	(3.2)	0.09	12/306	(3.9)	0.02	82/2123	(3.9)	0.03	
*RAS use*										
No	23/447	(5.2)		24/359	(6.7)		105/1817	(5.8)		
Yes	42/1428	(2.9)	0.03	9/155	(5.8)	0.75	46/1562	(2.9)	<0.001	

*Chi-square test, accounting for clustering at provider level

#### Illness

**Fig. 3:**
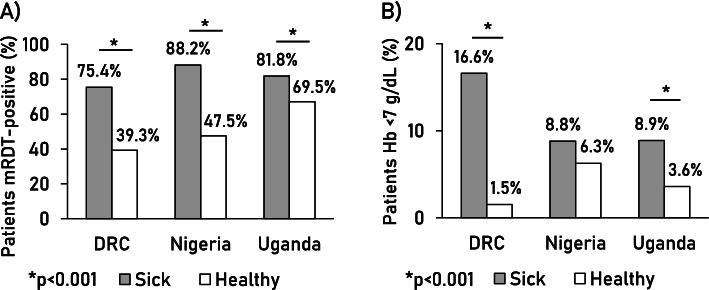
Percent of study patients with **A)** a positive mRDT and **B)** severe anaemia (Hb <7 g/dL) at 28 day follow-up, by health status

In addition, 865 patients were found to be sick on the day of follow-up, with a significant difference between the countries (*p* = 0.002) (Table 3; complementary data in Additional file 2: Figure S2). Conversely, a comparable proportion of patients was healthy (i.e. neither dead nor sick) in DRC (81%), Nigeria (83%), and Uganda (84%) (*p* = 0.54). Of 6020 patients tested by mRDT at follow-up, 44.0% were positive in DRC, 50.2% in Nigeria, and 69.5% in Uganda (Table 3). Those reported sick were significantly more likely to be mRDT-positive than those who were healthy (*p* < 0.001) (Fig. 3A). In the healthy group, HRP2/pLDH combo tests more frequently detected only HRP2 than in sick patients in DRC and Uganda (*p* < 0.001, Additional file 2: Figure S3), suggesting persistent antigenaemia rather than an active infection, as HRP2 may persist in a patient’s blood for a prolonged time period after parasite clearance [21]. Conversely, a substantial proportion of sick children in DRC (56.6%) and Nigeria (66.7%) had a pLDH-positive test, suggesting an active infection (Additional file 2: Figure S3).

Hb concentration measured in 5768 patients at follow-up found a lower mean Hb in Nigeria (9.9 g/dL) than in DRC and Uganda (both 10.3 g/dL, *p* < 0.001). Sick children in DRC and Uganda were more likely to have severe anaemia (Hb < 7 g/dL) than healthy children (both *p* < 0.001) (Fig. 3B and Additional file 2: Figure S4).

### Health impact of rectal artesunate

In DRC, there was no evidence of an association of RAS use with death by day 28 (OR = 1.25, 95% CI 0.81–1.93) (Table 4A). After adjusting for covariates including referral completion and post-referral treatment, however, RAS use appeared to have a negative effect on survival (aOR = 3.06, 95% CI 1.35–6.92). On the other hand, children who received RAS were more likely to receive both a parenteral antimalarial and ACT at a referral health facility (44.9% vs 22.1% of non-RAS-users, *p* < 0.001), and patients who received both of these medicines were less likely to die than those who received neither (aOR = 0.13, 95% CI 0.07–0.24). RAS use did not appear to reduce the likelihood of being dead or sick at follow-up (aOR = 0.88, 95% CI 0.59–1.32) (Table 4B). Patients in DRC who did not complete referral were less likely to be dead or sick at follow-up than those who completed referral within a day.

**Table 4:** Association of health outcome at day 28 follow-up and selected predictors, including pre-referral RAS treatment

	Covariate	OR	(95% CI)	Covariate	OR	(95% CI)	Covariate	OR	(95% CI)
**A) Dead at follow-up**	**DRC**			**Nigeria**			**Uganda**		
Unadjusted	RAS use	1.25	(0.81–1.93)	RAS use	2.95	(1.78–4.90)	RAS use	0.70	(0.29–1.74)
Adjusted^a^	RAS use	1.96	(0.88–4.35)	RAS use	2.57	(1.40–4.73)	n/a^c^		
Adjusted further for referral and post-referral treatment at RHF^a^	RAS use	3.06	(1.35–6.92)	RAS use	2.16	(1.11–4.21)	n/a^c^		
	*Referral*			*Referral*					
	Same or next day	Ref.		Same or next day	Ref.				
	Late	1.21	(0.71–2.07)	Late, not completed, unknown	1.58	(0.72–3.47)			
	Not completed	0.20	(0.10–0.39)						
	Unknown	0.82	(0.33–2.02)						
	*Treatment*			*Treatment*					
	Inj. AS/ART/QU only	Ref.		Inj. AS/ART/QU only	Ref.				
	ACT only	0.36	(0.07–1.70)	ACT only	n/a				
	Both	0.13	(0.07–0.24)	Both	n/a				
	None	1.14	(0.56–2.33)	None	5.85	(1.06–32.35)			
	Unknown	2.07	(1.10–3.90)	Unknown	7.65	(2.72–21.53)			
**B) Dead or sick at follow-up**	**DRC**			**Nigeria**			**Uganda**		
Unadjusted	RAS use	0.84	(0.65–1.08)	RAS use	1.95	(1.27–3.01)	RAS use	0.40	(0.33–0.49)
Adjusted^b^	RAS use	0.75	(0.51–1.13)	RAS use	1.62	(1.01–2.58)	RAS use	0.57	(0.43–0.76)
Adjusted further for referral and post-referral treatment at RHF^b^	RAS use	0.88	(0.59–1.32)	RAS use	1.42	(0.85–2.36)	RAS use	0.60	(0.45–0.79)
	*Referral*			*Referral*			*Referral*		
	Same or next day	Ref.		Same or next day	Ref.		Same or next day	Ref.	
	Late	0.93	(0.67–1.30)	Late	3.20	(1.21–8.42)	Late	0.85	(0.55–1.32)
	Not completed	0.36	(0.22–0.60)	Not completed	2.39	(1.08–5.29)	Not completed	0.74	(0.60–0.91)
	Unknown	0.82	(0.46–1.47)	Unknown	1.28	(0.55–2.97)	Unknown	0.93	(0.55–1.58)
	*Treatment*			*Treatment*			*Treatment*		
	Inj. AS/ART/QU only	Ref.		Inj. AS/ART/QU only	Ref.		Inj. AS/ART/QU only	Ref.	
	ACT only	0.28	(0.10–0.75)	ACT only	n/a		ACT only	1.06	(0.68–1.64)
	Both	0.40	(0.29–0.56)	Both	n/a		Both	0.52	(0.33–0.83)
	None	0.89	(0.53–1.52)	None	4.58	(0.97–21.59)	None	1.34	(0.77–2.33)
	Unknown	1.11	(0.68–1.80)	Unknown	3.26	(1.41–7.51)	Unknown	1.47	(0.96–2.26)

*ACT* Artemisinin-based combination therapy, *AS* Artesunate, *ART* Artemether, *n/a* not applicable, *QU* Quinine, *RAS* Rectal artesunate

^a^Fixed effects, DRC: sex, age <1 year, beginning of RAS roll-out, convulsions, enrolment location (CHW vs. PHC), rainy season; Nigeria: convulsions, enrolment location (CHW vs. PHC); random effect: enrolling provider (all countries)

^b^Treatment coded ‘unknown’ for patients who did not complete referral; fixed effects, DRC: sex, age <1 year, beginning of RAS roll-out, convulsions, enrolment location (CHW vs. PHC), rainy season, health zone, Nigeria: convulsions, enrolment location (CHW vs. PHC); Uganda: sex, age <1 year, beginning of RAS roll-out, convulsions, rainy season, district; random effect: enrolling provider (all countries)

^c^No adjusted models were calculated due to an insufficient number of events (death)

In Nigeria, patients who had received RAS were significantly more likely to die within 28 days (OR = 2.95, 95% CI 1.78–4.90) (Table 4A). Generally, the risk of dying was much higher among those enrolled at a PHC compared to a CHW (CFR = 18.5% vs. 5.7%, *p* = 0.001) (Additional file 2: Figure S5). Adjusted for covariates including referral completion and post-referral treatment, RAS use remained strongly associated with death (aOR = 2.16, 95% CI 1.11–4.21) while administration of a parenteral antimalarial was associated with survival (aOR = 0.17, 95% CI 0.03–0.95). Referral completion was not associated with CFR. With the same adjustments, there was no evidence of a significant association between RAS use and being dead or sick at follow-up (aOR = 1.42, 95% CI 0.85–2.36) but of a negative effect of delaying or not completing referral (Table 4B).

In Uganda, where the case fatality was lowest, no association was found between RAS use and death (OR = 0.70, 95% CI 0.29–1.74) (Table 4A). However, patients who received RAS were significantly less likely to be dead or sick at follow-up (aOR = 0.60, 95% CI 0.45–0.79) as were those who were treated with both a parenteral antimalarial and an ACT (aOR = 0.52, 95% CI 0.33–0.83). Patients in Uganda who did not complete referral were less likely to be dead or sick at follow-up than those who did so promptly (Table 4B).

Restricting these analyses to the time before COVID-19 measures were implemented in some areas (April 2020) did not change the observed effect or effect size of RAS use (Additional file 1: Table S2).

## Discussion

The current recommendation to use RAS as pre-referral treatment where parenteral alternatives are unavailable is based on a randomised controlled trial that provided little evidence of the effect of introducing RAS at scale [7–9, 22]. Other studies were implemented as intervention packages including both RAS and a strong support to referral mechanisms making it impossible to identify the contribution of RAS to improvements in health outcomes [23–25]. The CARAMAL Project represents the first large-scale assessment of the health impact of introducing RAS in existing community-level health care systems accompanied by only minimal interventions in support of the entire continuum of care [14], reflecting realistic scenarios of the anticipated large-scale roll-out of RAS [12]. Based on a systematic 28 days patient follow-up, our analysis provides robust evidence of the health impact of this intervention in three distinct sub-Saharan African settings with a high burden of malaria, and of health system factors that may promote or hamper the effectiveness of RAS as part of paediatric severe malaria care.

The beneficial effect of RAS pre-referral treatment on survival found in the trial by Gomes et al. [7, 8] could not be replicated in the ‘real-world’ scenarios of the three study sites. On the contrary, in DRC and Nigeria, patients treated with RAS were more likely to die over the course of the follow-up period. The use of RAS was found to have a slightly positive health effect only in Uganda, driven primarily by a reduction in children reported sick at follow-up (aOR = 0.61). Patients who were sick at follow-up had a positive mRDT or severe anaemia (Hb < 7 g/dL) more often than those who were healthy. Particularly in DRC and Nigeria, where mRDTs were frequently pLDH-positive in children who were sick on day 28, the findings suggest incomplete cure of the initial severe malaria episode (or a recent re-infection). RAS users were less often mRDT-positive (DRC) or severely anaemic (DRC and Uganda) at follow-up.

The increased CFR associated with the roll-out and use of RAS observed in DRC and Nigeria is likely a result of complex interactions between disease severity, treatment seeking, and care provided in the context of weak health systems, rather than a direct result of RAS treatment which was previously shown to be safe and efficacious [26, 27]. Secular trends in disease incidence and severity may have played a confounding role in DRC, where a larger number of severe cases were enrolled in the post-RAS period (Additional file 2: Figure S5). In Nigeria, CFR was highest in children enrolled at PHCs (Additional file 2: Figure S5) and patients attending PHCs were found to be more severely ill than those seen by a CHW [28]; the regression model was therefore adjusted for enrolment location. Concomitant infections and septicaemia may have contributed to the increased CFR. For example, Lassa fever is known to occur in the study area but often remains undiagnosed due to unspecific symptoms and lack of diagnostic facilities [29]. A small number of cases were reported from Adamawa State in 2020 [30] but none of the study patients was formally diagnosed with Lassa fever or another severe viral infection. In the period after the roll-out of RAS, patients in Nigeria enrolled during the COVID-19 pandemic were less likely to complete referral than those enrolled earlier [19]. Yet, while COVID-19 pandemic measures may have influenced treatment seeking or provision of care, limiting the health outcome analyses to the pre-Covid-19 period did not change the observed effect of RAS. In Uganda, a country-wide increase in malaria was reported in 2019, overlapping with the early RAS implementation phase (NMCP, personal communication). Anecdotal evidence suggests that health workers stocking only a small number of RAS doses may have administered RAS preferentially to more severely sick children. While the small number of deaths in Uganda did not allow accounting for such potential confounders in the CFR analysis, an imbalance between RAS users and non-users in the frequency of convulsion, a symptom often associated with cerebral malaria [18, 31], was adjusted for in all other analysis. Due to the community-level enrolment strategy in all countries, an expert clinical assessment of patients (incl. diagnosis of co-morbidities) was not available.

In the absence of comprehensive measures to strengthen the underlying health system implemented synergistically with the introduction of RAS [32], several factors along the continuum of care are likely to have hampered the effectiveness of RAS. Between 35 and 48.7% of study patients did not complete referral to an appropriate referral health facility. In DRC and Nigeria, we found evidence of an adverse effect of RAS use on referral completion [19]. Non-completion of referral after pre-referral treatment with RAS, possibly due to an initial improvement of the child’s condition, has been reported from other studies [25, 33]. Failing to attend a referral health facility may result in patients not obtaining adequate comprehensive treatment for their severe illness episode. Economic barriers and distance were frequently mentioned as reasons for not completing referral in this study (unpublished data, CARAMAL Project). The finding that patients in DRC who did not complete referral were less likely to die may be a result of less severely sick patients, or those recovering quickly after a dose of RAS, not being brought to a referral facility. This is supported by the finding that patients perceived not to be fatally ill were significantly less likely to complete referral [19]. Improved health has already previously been reported as a reason for non-compliance with referral advice [25, 34].

Comprehensive antimalarial treatment after a dose of RAS is crucial as one dose of artesunate alone (or in combination with another only partly effective antimalarial) cannot fully clear an infection [2, 35]. This study found patients receiving post-referral treatment with a parenteral antimalarial plus ACT to be significantly less likely to die (or be sick at follow-up). While being a plausible result, the calculated coefficients are unlikely to reflect the true effect size as survival is a condition for receiving post-referral treatment. Yet, many patients who were admitted with severe malaria in the study areas of all three countries did not receive an ACT at the referral facility after treatment with parenteral antimalarials [20]. In Nigeria, parenteral treatment was common, but none of the patients received an ACT at the Cottage Hospitals. Whether ACT treatment courses that were merely prescribed upon discharge at referral facilities were actually purchased and administered could not be verified for all patients in this study. Pre-referral treatment with RAS with or without subsequent parenteral artesunate, but without an oral ACT, constitutes an artemisinin monotherapy treatment. This is a risk for artemisinin resistance development and positive selection of circulating resistant parasites, the latter of which has already been documented in four African countries including Uganda [36, 37]. Consolidated action is required to improve compliance with treatment guidelines that require a full course of oral ACT to follow parenteral treatment and to establish routine artemisinin resistance monitoring across African malaria-endemic countries to detect and prevent the further spread of artemisinin resistant parasites.

All patients included in this study had access to formal health care providers (enrolment criterion) and 42–73% of deaths occurred in a health facility, often several days after first contact with the formal health system (mean 2.4–6.3 days). In Nigeria, where ACTs were not provided as post-referral treatment, 88% of those who were sick on day 28 had a positive mRDT (mostly pLDH-positive), and the CFR among patients enrolled at a PHC exceeded 20% over several months (Additional file 2: Figure S5). While a comprehensive assessment of the quality of care provided in referral health facilities was beyond the scope of this study, together, these findings reflect weak health care systems that are often unable to save the lives of severely sick children. In these challenging settings, pre-referral RAS did not appear to have a beneficial health effect despite the potential of this intervention demonstrated under different circumstances [7]. Further investigations of the treatment and care provided after pre-referral RAS administration may help to identify specific improvements along the continuum of care that are essential to save the lives of severely sick children. These lessons, learned during the implementation of pre-referral RAS and documented here and elsewhere [14, 19, 20], may be similarly relevant for the large-scale roll-out of other malaria control interventions, including the malaria vaccine RTS,S/AS01 that was recently recommended by the WHO [38]. The decay in the effectiveness of health interventions implemented in complex systems should therefore be carefully evaluated alongside, or better prior to, their large-scale implementation [39].

Introducing pre-referral RAS without guaranteeing an effective continuum of care with prompt referral and high-quality post-referral case management (including the diagnosis and appropriate management of co-morbidities and complications) is unlikely to result in a decrease in mortality in settings with a high malaria burden but a weak health system. Conversely, RAS is most likely to be beneficial in locations where peripheral health care is provided by lay health workers, but post-referral services are available, accessible, and of good quality. This is supported by the finding of a moderately beneficial effect in Uganda, where baseline CFR was lowest, the number of CHW per person was highest, accessibility of formal health facilities was least problematic (measured, e.g. in distance/time to facility), and out of pocket treatment costs were lowest [32]. Evidence from implementing a package of interventions including RAS in Zambia also supports this notion [23].

## Conclusions

Curbing the remaining burden of malaria mortality remains a top public health priority in countries with a high malaria transmission. Pre-referral RAS treatment may have a beneficial health effect for an individual patient who follows the entire continuum of care. Yet, the intervention is unlikely to reduce malaria mortality in a population unless underlying health system weaknesses are addressed. The large-scale roll-out of pre-referral RAS must be accompanied by measures to ensure definitive treatment with at least parenteral artesunate and a full course of oral ACT in higher-level health facilities.

## Supplementary Information

**Additional file 1: Table S1**. Study settings. **Table S2.** Adjusted regression estimates, overall and restricted to pre-COVID-19 period (before April 2020).

**Additional file 2: Figure S1.** Inclusion flow-charts. **Figure S2.** Complementary day 28 health outcome indicators, by country. **Figure S3.** Results of HRP2/pLDH Combo tests at day 28 follow-up, by detected antigen (HRP2 and/or pLDH), in a sub-sample of study patients. **Figure S4.** Mean haemoglobin (Hb) concentration and percent of children with anaemia (Hb <11 g/dL) among children sick or healthy at follow-up. **Figure S5.** (A) Time trend in monthly inclusions and case fatality ratio (CFR) expressed as 3-month moving average, by type of enrolling provider, and (B) overall CFR by enrolling provider.

## Data Availability

De-identified individual participant data that underlie the results reported in this article are available at zenodo.org (DOI: 10.5281/zenodo.5548261) and access is provided upon reasonable request [40].

## Abbreviations

ACT: Artemisinin-based combination therapy
aOR: Adjusted odds ratio
CARAMAL: Community Access to Rectal Artesunate for Malaria
CFR: Case fatality rate
CHW: Community health worker
CI: Confidence interval
DRC: Democratic Republic of the Congo
Hb: Haemoglobin
iCCM: Integrated Community Case Management
mRDT: Malaria rapid diagnostic test
OR: Odds ratio
PHC: Primary health centre
RAS: Rectal artesunate
RR: Risk ratio
UNICEF: United Nations Children’s Fund
WHO: World Health Organization
