# Supplementary material for: Effectiveness of rectal artesunate as pre-referral treatment for severe malaria in children under 5 years of age: a multi-country observational study
Source: BMC Med. 2022 Oct 11;20:343. doi: 10.1186/s12916-022-02541-8 (PMC9550309; doi:10.1186/s12916-022-02541-8)
Supplement: Supplementary file 1 — Additional file 1: Table S1. Study settings. Table S2. Adjusted regression estimates, overall and restricted to pre-COVID-19 period (before April 2020). [file 12916_2022_2541_MOESM1_ESM.pdf]

Table S1: **Study settings**

	**DRC**	**Nigeria**	**Uganda**
**Population (children <5 years)**			
	Health Zone	*Local Government Area*	*District*
	Ipamu: 284’484 (52’523)	Fufore: 280’660 (49’982)	Kole: 298’394 (59’184)
	Kenge: 382’232 (70’567)	Mayo-Belwa: 204’746 (33’101)	Kwania: 232’402 (48’582)
	Kingandu: 119’251 (22’016)	Song: 261’543 (47’347)	Oyam: 465’190 (92’752)
	Total: 785’968 (145’107)	Total: 746’949 (130’430)	Total: 995’986 (200’518)
Source	https://www.worldpop.org		
**Public health care providers***			
Community Health Workers	Site de Soin Communautaire (Community Care Site) (N = 42)	Community-Oriented Resource Person (CORP) (N = 500)	Village Health Team (VHT) members (N = 5,100)
Primary Health Care	Poste de Santé (Health Post) Centre de Santé (Health Centre) (N = 152)	Health Post** Primary Health Centre (PHC) (N = 77)	Health Centre II (HC II) (N = 30)
Referral Facilities	Centre de Santé de Référence (Referral Health Centre) and Hôpital Général de Référence (General Referral Hospital) (N = 19)	Cottage Hospital (N = 3)	Health Centre III (HC III), Health Centre IV (HC IV) and Hospital (N = 20)
**General danger signs as per national iCCM guidelines**			
	- Unusually sleepy or unconscious<br>- Not able to drink or feed anything<br>- Vomiting everything<br>- Convulsions	- Unusually sleepy or unconscious<br>- Not able to drink or feed anything<br>- Vomiting everything<br>- Convulsions<br>- Not responding to ACT<br>- Yellowness of the eyes	- Very sleepy or unconscious<br>- Not able to breastfeed or drink<br>- Vomiting everything<br>- Convulsions

*Number of providers at the beginning of the study period. **Health posts were upgraded to Primary Health Centres during the study period.

Table S2: **Adjusted regression estimates, overall and restricted to pre-Covid-19 period (before April 2020)**

**A) Dead at follow-up**	**DRC**			**Nigeria**			**Uganda**		
	**Covariate**	**aOR**	**(95% CI)**	**Covariate**	**aOR**	**(95% CI)**	**Covariate**	**OR**	**(95% CI)**
RAS use adjusted for basic covariates* and referral and treatment	RAS use	3.06	(1.35-6.92)	RAS use	2.16	(1.11-4.21)	RAS use§	0.70	(0.29-1.74)
	Pre-Covid-19 only: #			*Pre-Covid-19 only:* #			*Pre-Covid-19 only:* #		
	RAS use	3.32	(1.39-7.92)	RAS use	2.18	(0.90-5.27)	RAS use§	0.75	(0.29-1.93)
**B) Dead or sick at follow-up**	**DRC**			**Nigeria**			**Uganda**		
	**Covariate**	**aOR**	**(95% CI)**	**Covariate**	**aOR**	**(95% CI)**	**Covariate**	**aOR**	**(95% CI)**
RAS use adjusted for basic covariates** and referral and treatment	RAS use	0.88	(0.59-1.32)	RAS use	1.42	(0.85-2.36)	RAS use	0.60	(0.45-0.79)
	Pre-Covid-19 only: #			*Pre-Covid-19 only:* #			*Pre-Covid-19 only:* #		
	RAS use	0.84	(0.55-1.29)	RAS use	1.45	(0.78-2.68)	RAS use	0.64	(0.48-0.88)

*Fixed effects, DRC: sex, age <1 year, beginning of RAS roll-out, convulsions, enrolment location (CHW vs. PHC), rainy season; Nigeria: convulsions, enrolment location (CHW vs. PHC); random effect: enrolling provider (all countries)

**Fixed effects, DRC: sex, age <1 year, beginning of RAS roll-out, convulsions, enrolment location (CHW vs. PHC), rainy season, health zone, Nigeria: convulsions, enrolment location (CHW vs. PHC); Uganda: sex, age <1 year, beginning of RAS roll-out, No. danger signs (1-2, 3-4), rainy season, district; random effect: enrolling provider (all countries)

§No adjusted models were calculated due to insufficient number of events (death)

#Pre-Covid-19: DRC N = 1548, Nigeria N = 475, Uganda N = 3268. RAS = rectal artesunate
