# Supplementary material for: Effectiveness of rectal artesunate as pre-referral treatment for severe malaria in children under 5 years of age: a multi-country observational study
Source: BMC Med. 2022 Oct 11;20:343. doi: 10.1186/s12916-022-02541-8 (PMC9550309; doi:10.1186/s12916-022-02541-8)
Supplement: Supplementary file 2 — Additional file 2: Figure S1. Inclusion flow-charts. Figure S2. Complementary day 28 health outcome indicators, by country. Figure S3. Results of HRP2/pLDH Combo tests at day 28 follow-up, by detected antigen (HRP2 and/or pLDH), in a sub-sample of study patients. Figure S4. Mean haemoglobin (Hb) concentration and percent of children with anaemia (Hb <11 g/dL) among children sick or healthy at follow-up. Figure S5. (A) Time trend in monthly inclusions and case fatality ratio (CFR) expressed as 3-month moving average, by type of enrolling provider, and (B) overall CFR by enrolling provider. [file 12916_2022_2541_MOESM2_ESM.pdf]

Figure S1: **Inclusion flow-charts**

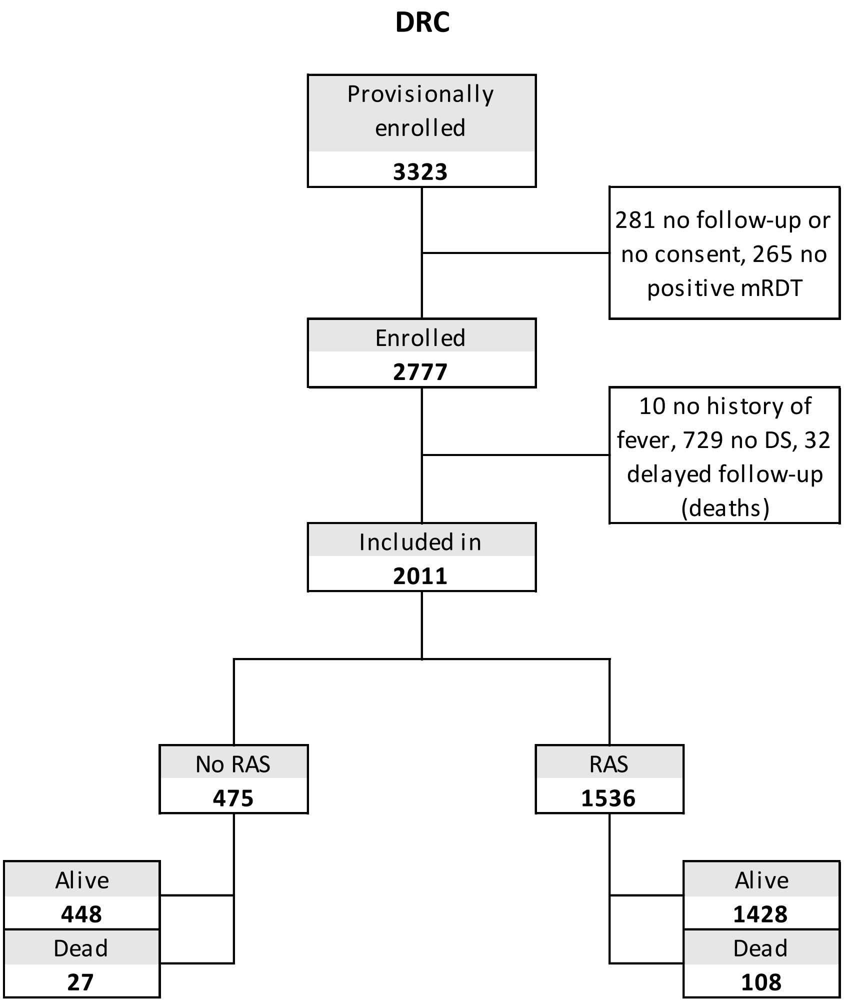

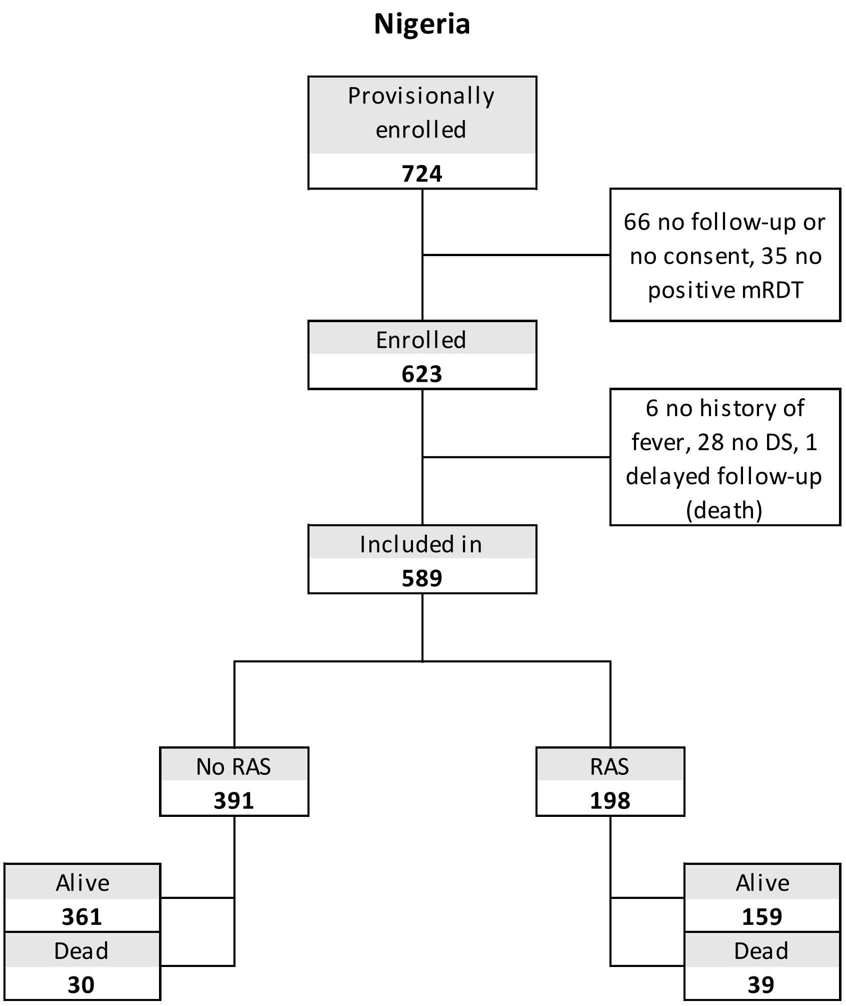
Nigeria
Provisionally enrolled
724
66 no follow-up or no consent, 35 no positive mRDT
Enrolled
623
6 no history of fever, 28 no DS, 1 delayed follow-up (death)
Included in
589
No RAS
391
RAS
198
Alive
361
Dead
30
Alive
159
Dead
39

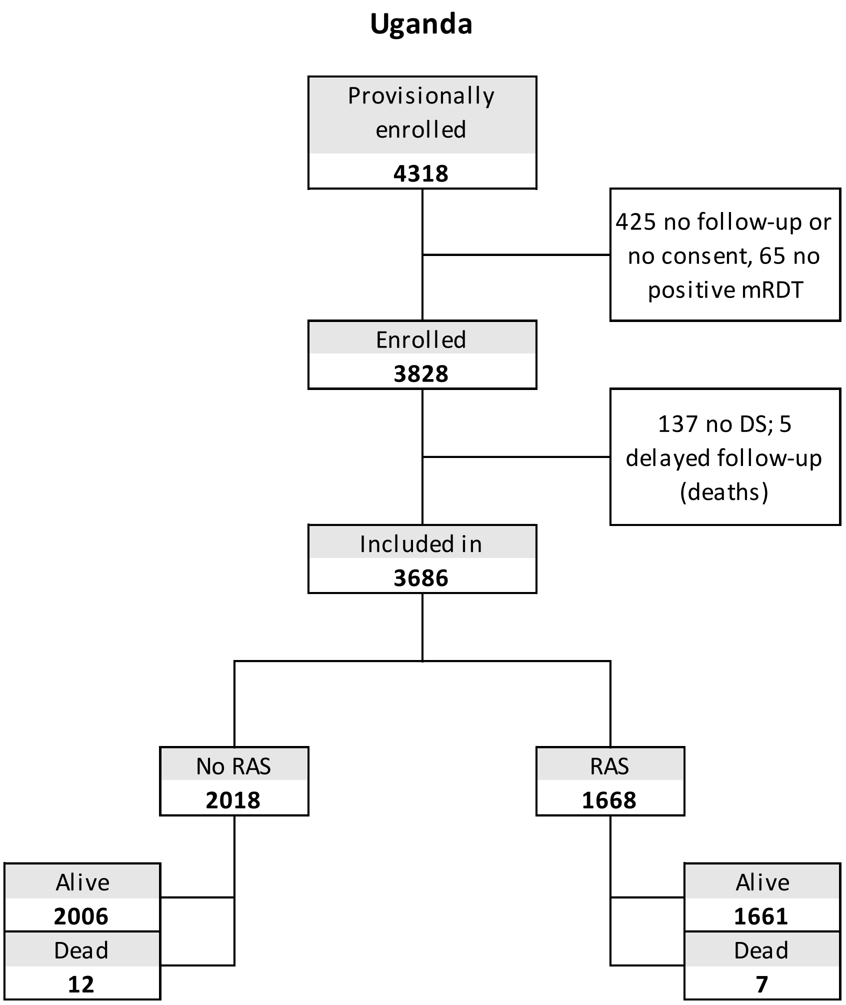
Uganda
Provisionally enrolled
4318
425 no follow-up or no consent, 65 no positive mRDT
Enrolled
3828
137 no DS; 5 delayed follow-up (deaths)
Included in
3686
No RAS
2018
RAS
1668
Alive
2006
Dead
12
Alive
1661
Dead
7

Figure S2: **Complementary day 28 health outcome indicators, by country**

A)

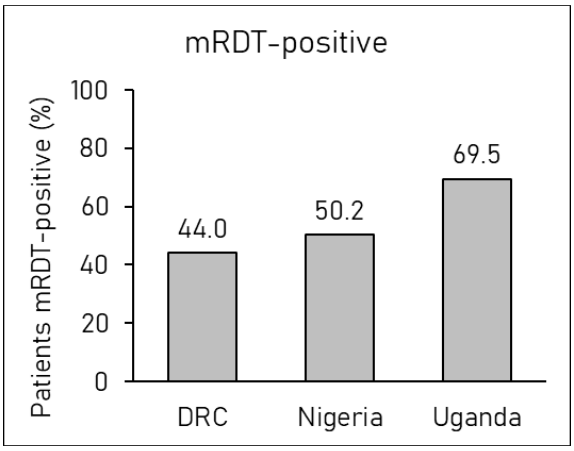

B)

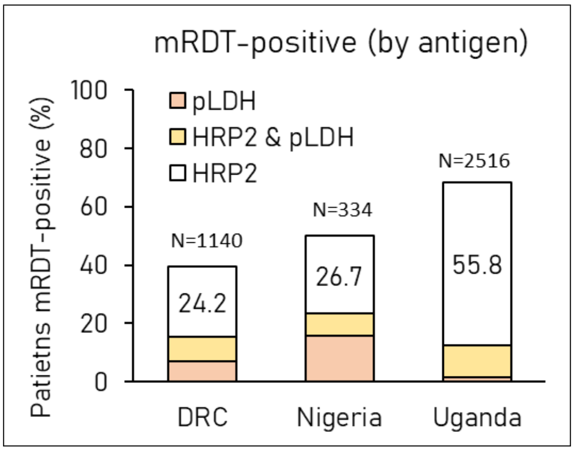

C)

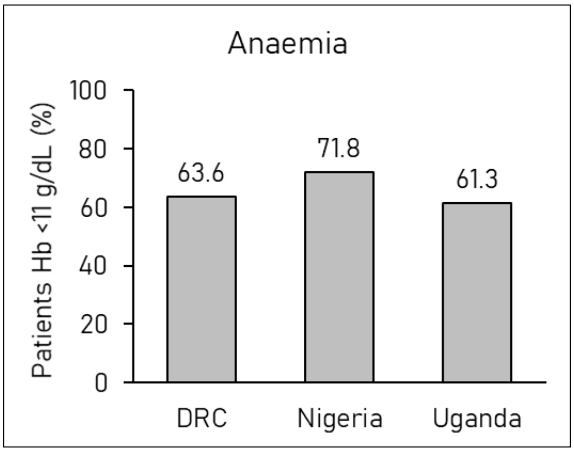

D)

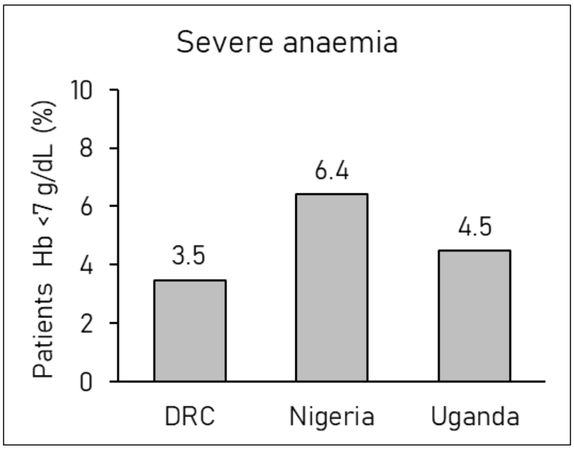

E)

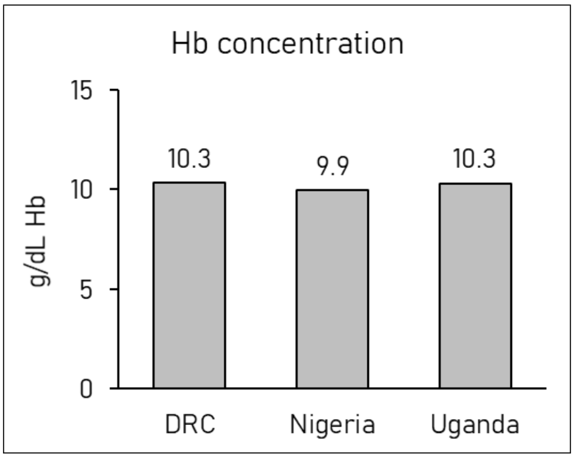

Reference for definitions of anaemia and severe anaemia in C) and D): WHO. Haemoglobin concentrations for the diagnosis of anaemia and assessment of severity. Vitamin and Mineral Nutrition Information System. Geneva, World Health Organization, 2011 (WHO/NMH/NHD/MNM/11.1).

Figure S3: **Results of HRP2/pLDH Combo tests at day 28 follow-up, by detected antigen (HRP2 and/or pLDH), in a sub-sample of study patients**

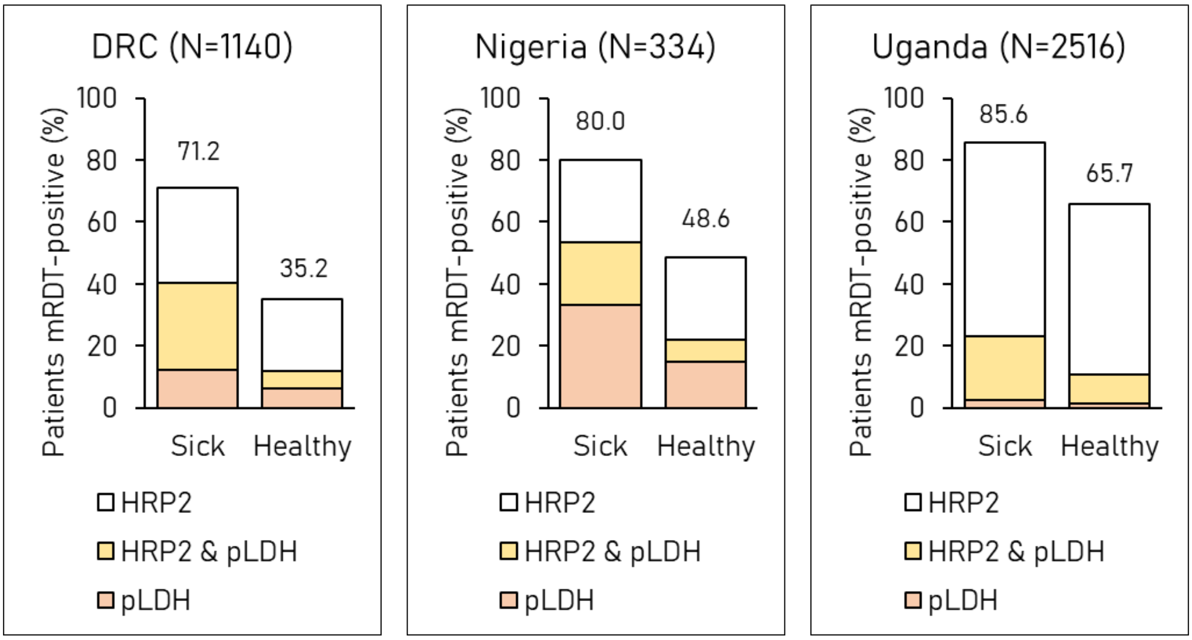

Proportion HRP2 only out of all with positive mRDT

DRC: healthy 66.2% vs. sick 43.4%, $p < 0.001$

Nigeria: healthy 54.8% vs. sick 33.3%, $p = 0.15$

Uganda: healthy 83.4% vs. sick 73.0%, $p < 0.001$

Figure S4: **Mean haemoglobin (Hb) concentration and percent of children with anaemia (Hb <11 g/dL) among children sick or healthy at follow-up**

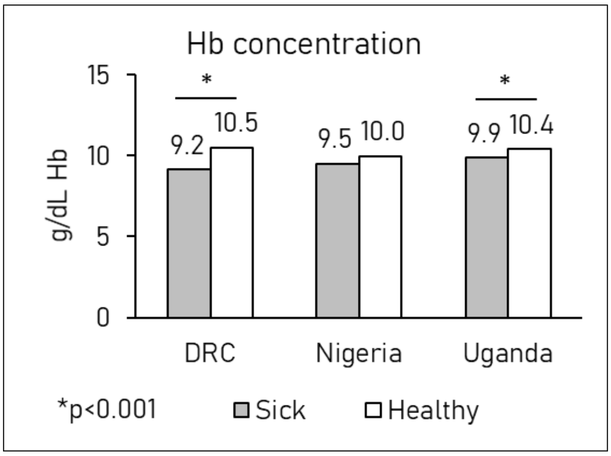

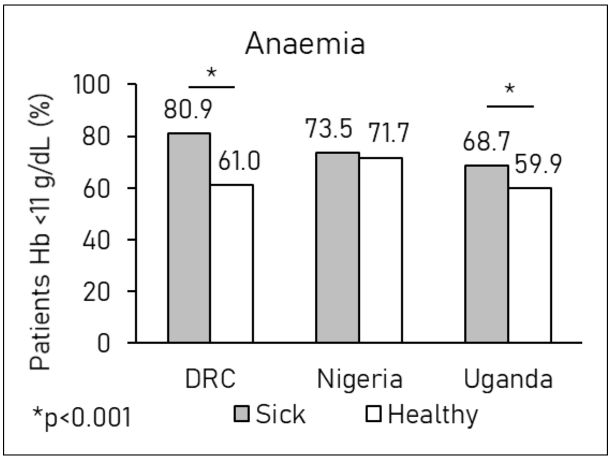

Reference for definitions of anaemia and severe anaemia: WHO. Haemoglobin concentrations for the diagnosis of anaemia and assessment of severity. Vitamin and Mineral Nutrition Information System. Geneva, World Health Organization, 2011 (WHO/NMH/NHD/MNM/11.1).

Figure S5: **(A) Time trend in monthly inclusions and case fatality ratio (CFR) displayed as 3-month moving average, by type of enrolling provider, and (B) overall CFR by enrolling provider**

DRC

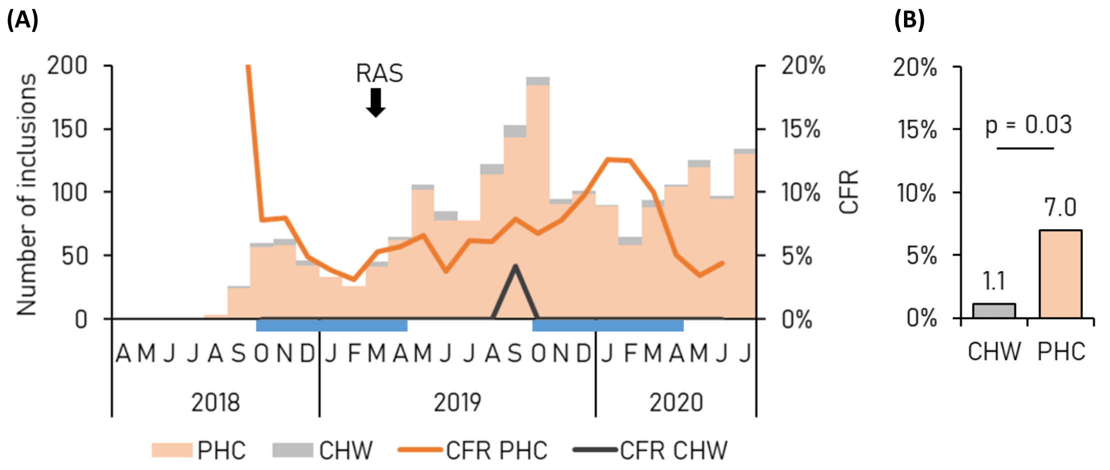

Nigeria

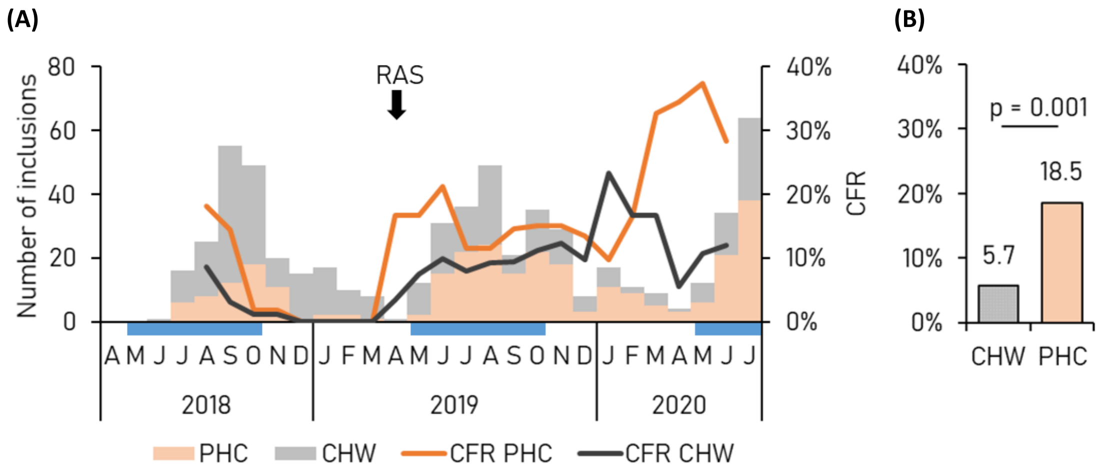

Uganda

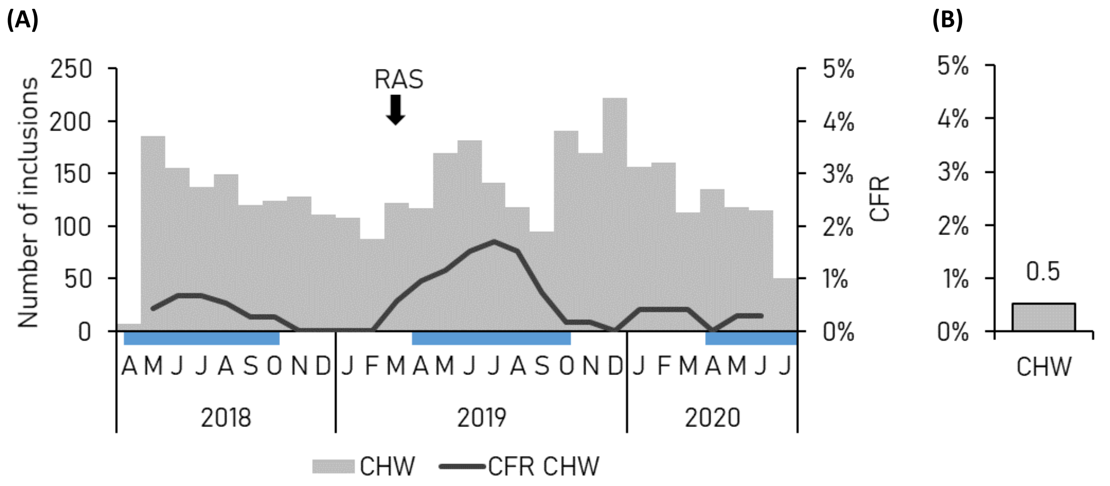

CHW = community health worker, PHC = primary health centre

Horizontal blue bars denote the rainy season
